# A Systematic Review of Social Factors and Suicidal Behavior in Older Adulthood

**DOI:** 10.3390/ijerph9030722

**Published:** 2012-03-01

**Authors:** Madeleine Mellqvist Fässberg, Kimberly A. van Orden, Paul Duberstein, Annette Erlangsen, Sylvie Lapierre, Ehud Bodner, Silvia Sara Canetto, Diego De Leo, Katalin Szanto, Margda Waern

**Affiliations:** 1 Department of Psychiatry and Neurochemistry, Neuropsychiatric Epidemiology Unit, Institute of Neuroscience and Physiology, Sahlgrenska Academy, University of Gothenburg, Wallinsgatan 6, 43141 Mölndal, Sweden; Email: madeleine.mellqvist@neuro.gu.se; 2 Department of Psychiatry, University of Rochester Medical Center, 300 Crittenden Boulevard, Rochester, NY 14642, USA; Email: Kimberly_Vanorden@URMC.Rochester.edu (K.A.O.); Paul_Duberstein@URMC.Rochester.edu (P.D.); 3 Department of Mental Health, Johns Hopkins School of Public Health, 624 North Broadway, S850, Baltimore, MD 21205, USA; Email: aerlangs@jhsph.edu; 4 Département de Psychologie, Université du Québec à Trois-Rivières, 3351 des Forges blvd., Trois-Rivières, QC G9A 5H7, Canada; Email: Sylvie.Lapierre@uqtr.ca; 5 The Interdisciplinary Department of Social Sciences and the Music Department, Bar-Ilan University, Ramat-Gan, Israel; Email: Ehud.Bodner@biu.ac.il; 6 Department of Psychology, Colorado State University, Fort Collins, CO 80523, USA; Email: Silvia.Canetto@colostate.edu; 7 Australian Institute for Suicide Research and Prevention, National Centre of Excellence in Suicide Prevention, WHO Collaborating Centre for Research and Training in Suicide Prevention, and Life Promotion Clinic, Mt Gravatt Campus, Griffith University,176 Messines Ridge Road, Mt Gravatt, QLD 4122, Australia; Email: d.deleo@griffith.edu.au; 8 Western Psychiatric Institute and Clinic, University of Pittsburgh, 3811 O’Hara Street, Pittsburgh, PA 15260, USA; Email: SzantoK@upmc.edu; 9 Department of Psychiatry and Neurochemistry, Sahlgrenska University Hospital, Sahlgrenska Academy, University of Gothenburg, Blå Stråket 15, 41345 Gothenburg, Sweden

**Keywords:** death wishes, suicidal ideation, non-fatal suicidal behavior, suicide, social factors, social support, systematic review, older adults

## Abstract

Suicide in later life is a global public health problem. The aim of this review was to conduct a systematic analysis of studies with comparison groups that examined the associations between social factors and suicidal behavior (including ideation, non-fatal suicidal behavior, or deaths) among individuals aged 65 and older. Our search identified only 16 articles (across 14 independent samples) that met inclusion criteria. The limited number of studies points to the need for further research. Included studies were conducted in Canada (n = 2), Germany (n = 1), Hong Kong (n = 1), Japan (n = 1), Singapore (n = 1), Sweden (n = 2), Taiwan (n = 1), the U.K. (n = 2), and the U.S. (n = 3). The majority of the social factors examined in this review can be conceptualized as indices of positive social connectedness—the degree of positive involvement with family, friends, and social groups. Findings indicated that at least in industrialized countries, limited social connectedness is associated with suicidal ideation, non-fatal suicidal behavior, and suicide in later life. Primary prevention programs designed to enhance social connections as well as a sense of community could potentially decrease suicide risk, especially among men.

## 1. Introduction

Suicide in later life is a global public health problem, with those aged 65 and above constituting the demographic group with the highest suicide rate in most countries that report suicide statistics to the World Health Organization [1]. Countries with high rates of older adult suicide include European Union countries, Canada, the U.S., and several Asian countries, including Japan, Singapore, and Taiwan. Older men are at particularly elevated risk in these countries [1,2], with the gender gap in suicide mortality less pronounced in Asian countries [3]. Prevention of late-life suicide is marked by numerous challenges. First and foremost, in many countries older adult suicidal behavior is highly lethal [2]. Non-fatal suicidal acts tend to be less common in this age group [4]. For example, in the U.S., up to 75% of older adults die as a consequence of their first suicidal act [5], which may be explained by age-related increases in planning [6], physical frailty and method lethality [7]. Further, U.S. older persons are less likely to report thoughts of suicide to others [8] and to use mental health services [9,10]. Thus, interventions are needed to prevent suicidal behavior also among those with no prior history of suicidal behavior and those who do not seek psychiatric treatment. 

Psychological autopsy studies (in which detailed psychiatric, medical and psychosocial histories are obtained for individuals who died by suicide via structured interviews with family and others who knew the individuals well) indicate that mental disorder is present in 71–97% of older adults who die by suicide with depression as the most common diagnosis [2]. A U.S.-based study demonstrated that depression is a particularly strong correlate of suicide in later life [11]. Thoughts of suicide become less prominent or frequent when depression remits in older persons who are treated for depression [12]. Preventing or treating depression could thus constitute powerful suicide prevention strategies for this age group. The possible preventive role of depression screening and follow-up was highlighted via a meta-analysis of quasi-experimental studies of older adults in several Japanese regions. Reduced suicide rates were observed in the intervention regions, particularly in women [13]. Collaborative care models (CCM) for depression which involve augmenting depression treatment in primary care with expert mental health consultation and medication recommendations, psychoeducation for patients, and the option of brief psychotherapy have been shown to reduce suicide ideation among older adults in the U.S. [14,15]. However, suicide ideation may persist following treatment in some individuals. Further, decreases in the frequency or severity of thoughts about suicide do not necessarily indicate reduced suicide risk. Most depressed people do not die by suicide, and there are modifiable risk markers for both depression and suicide that can be targeted before the emergence of depression, suicidal feelings, and other risk factors for suicide mortality. Social factors can constitute important targets in this context. 

The influential role of social factors in the etiology of suicide was highlighted by Durkheim [16], whose model of suicide focuses on two social forces, social integration and moral integration. According to Durkheim, changes in suicide rates at the societal level will occur when these forces become dysregulated (*i.e.*, too strong or too weak). Specifically, too much or too little social integration as well as too much or too little moral integration should be associated with increased suicide rates according to Durkheim, due to the emergence of subtypes of suicide, each with a distinct social factor underlying the etiology. In the case of too much social integration, Durkheim proposed that “altruistic” suicides result because members of society become willing to sacrifice themselves for the larger good. Regarding too little social integration, Durkheim proposed that “egoistic” suicides result because members of society do not feel connected to their society—to something that transcends their individual experiences and provides an existential anchor. In the case of too much moral integration Durkheim proposed that “fatalistic” suicides result because members of society experience a total lack of autonomy due to societal over-regulation of beliefs and behaviors through such factors as social norms or the justice system). Regarding too little moral integration, Durkheim proposed that “anomic” suicides result because members of society lack a sense of security and structure. 

Psychological theorists have also proposed causal roles for social factors, but in contrast to sociological theorists (e.g., Durkheim), they emphasize connections at the individual level, rather than connections with society. Most of these theories focus on familial relationships. Feeling isolated from family members, experiencing family discord, and perceiving oneself to be a burden on family members are all social factors posited by psychological theorists to be involved in the etiology of suicide [17,18]. One psychological theory that does not specifically focus on the family is the Interpersonal Theory of Suicide [16], which proposes that individuals have a psychological need to feel connected to and cared about by others, termed the need to belong, and that when this need is completely unmet, a passive desire for death will develop. The theory also posits that when individuals perceive that they are a burden on others (regardless of the veracity of the belief) such that others would be better off if they were gone, a passive desire for death will develop. Further, the theory specifies that when the need to belong is unmet and perceptions of burdensomeness are present, a passive desire for death will transform into active thoughts of killing oneself. Finally, the Interpersonal Theory also proposes that social factors are not sufficient to explain suicide. Other psychological factors are needed to explain why the vast majority of those who think about suicide do not actually harm themselves. The theory proposes that this is the case because most individuals are not capable of suicide, and to be capable, individuals must lose some of the fear associated with suicidal behavior and be able to tolerate the pain that accompanies suicidal behavior. 

Common themes from these diverse theorists are that positive social connections may be protective against suicide, whereas discordant or overly strong/enmeshed connections may elevate risk for suicide. Another theme is that social factors alone are insufficient to explain the etiology of suicidal behavior. Rather, these social factors interact with characteristics of individuals to influence the risk for suicide. The social context is a crucial factor in understanding risk for suicide [19] and to make sense of the overwhelmingly high rates of suicide in older males, especially in U.S., Canada and many European countries [20]. In fact, the only randomized intervention trials with evidence suggesting a reduction of suicides, though not conducted specifically with older adults, have been posited to work by enabling at-risk individuals to feel more connected to others and cared for [21,22]. There are several quasi-experimental studies of interventions designed to increase social connectedness (*i.e.*, the degree to which older adults are connected to family, friends, and their communities) with results suggesting that this is a promising strategy for reducing late-life suicide rates [13,23,24]. While a role for social factors has been indicated in several reviews on suicide in later life [2,25], we are aware of no systematic review focusing specifically on social factors and suicidal behavior in this age group. Thus, the aim of this review was to identify social factors that are robustly associated with suicide and related phenomena among persons aged 65 and above. This review will address the following questions:

(1) Which social factors are associated with death wishes, suicide ideation, non-fatal suicidal behavior, and/or suicides in later life?
(2) What are areas in need of future research on social factors in late-life suicidal behavior?
(3) What are the implications for the prevention of suicidal behavior in this age group?

## 2. Method

Guidelines from the Cochrane Collaboration were used for this systematic review [26]. Case-comparison studies that focused on persons aged 65 years and above, published in peer-reviewed journals, and examining both social factors and suicide or related phenomenon (death wishes, suicide ideation, deliberate self harm/non-fatal suicidal behavior) were considered eligible for inclusion. For the purpose of this review, we chose to focus on social factors related to social connectedness and social inclusion. 

Studies were identified through electronic searches which were performed through the ERIC, Scopus, SUMMON, PubMed, and PsycINFO databases. Search terms used were: suicid* OR death wishes OR deliberate self harm. No search terms relating to social factors were applied. All publication years were considered. The search was carried out during August–September 2011.

The combined searches yielded 23,299 references. Where a title or an abstract appeared to describe a study that included older persons and data on both social factors and suicidal behavior, the full article was retrieved and examined for relevance. Studies lacking similar age comparison groups were excluded as were those involving persons under age 65. Further, articles written in languages other than English were excluded. Only 16 publications met the inclusion criteria, and these publications were based on data from a total of 14 studies. Associations confirmed in multivariate analyses were preferred over univariate. 

## 3. Results

Findings of the review are shown by social factor in Table 1 and summarized in box score format in Table 2. Seven studies (with eight publications) focused on death wishes/suicide ideation [27,28,29,30,31,32,33,34]. Factors related to self-harm/non-fatal suicidal behavior were examined in six studies [27,35,36,37,38,39]. Only three studies (with four publications) examined suicide/fatal suicidal behavior [38,40,41,42]. 

*Marital status* was examined in eleven studies. Not having a current partner was associated with death wishes in a Canadian community-based study by Lapierre [30]. Bartels studied correlates of passive death ideation and active suicide ideation in over 2,000 U.S. primary care patients with depression, anxiety or problematic alcohol use [28]; no association was found with marital status. In a clinical study set in Singapore, Tan found that marital status did not distinguish depressed patients with and without suicidal ideation [33]. Similarly, no association with marital status was observed in a U.S. study conducted by Raue which examined suicidal ideation (passive/active/none) in recipients of in-home nursing [31]. Yen reported that being single or widowed was associated with suicidal ideation in community dwelling older persons in a study from Taiwan [34]. Marital status did not differ in persons who reported death wishes/suicidal thoughts/non-fatal suicidal behavior compared to those without death wishes in Barnow’s population-based study set in Germany [27]. A Swedish study conducted by Wiktorsson focused on individuals aged 70 and above who were hospitalized in connection with a suicide attempt and a population-based comparison group [39]. Those who were treated in connection with a suicide attempt were more likely to be unmarried/not cohabitating. No such relationship was found in a Japanese study by Takahashi [37], nor in Tsoh’s study that was set in Hong Kong [38]. In a Scotland-based study, Conaghan observed that persons who were hospitalized in connection with a non-fatal suicidal act were statistically significantly *more often* married than those in the community-based comparison group [35]. Finally, Turvey presented data from a prospective U.S. population-based study showing no association between marital status and suicide [41]. 

*Living arrangement* was examined in most of the studies included in this review. Results were mixed. Regarding *living alone*, no association was seen with death ideation in the Bartels study [28]. Further, no relationship was observed with suicide ideation in that study, which was also the case in the study by Raue [31], and the Yen study from Taiwan [34].

No difference in proportions living alone could be shown in a British study by Dennis that compared a self-harm group and a depressed comparison group [36]. However, in a clinical study set in Japan, Takahashi found that persons with a history of suicidal behavior more often lived alone than other psychiatric inpatients [37]. Living alone was associated with increased risk of suicide attempt in the Swedish study conducted by Wiktorsson [39]. No such association was found, however in the Rubenowitz study that focused on suicide in the same region [40] and similar results were observed for men and women. Data from that study were examined further by Waern, who carried out separate analyses for younger (65–74 years) and older (75+) age groups and found no difference between the two groups with regards to living alone [42].

**Table 1:** Review findings summarized by social factor.

Social factor	Authors	Study type	Country	Subjects	Outcome	Measure of social factor	Strength of association
Marital status	Lapierre *et al.* (2011) [30]	Population-based	Canada	Community dwellers ^C^	Wish to die		No current partner *vs.* married: OR = 1.9 (95% CI = 1.3–2.9)^ ii^
	Bartels *et al.* (2002) [28]	Clinical	USA	Primary care patients with depression ^C^	Death ideation/Suicidal ideation		Marital status: NS
	Tan *et al.* (2008) [33]	Clinical	Singapore	Patients with major depression ^A^	Suicidal thinking		Marital status: NS
	Raue *et al.* (2007) [31]	Clinical	USA	Homecare patients ^B^	Suicidal ideation		Marital status: NS
	Yen *et al.* (2005) [34]	Population-based	Taiwan	Community dwellers ^B^	Suicidal ideation		Single/widowed *vs.* married: OR = 2.04 (95% CI = 1.25–3.31) ^iii^
	Barnow *et al.* (2004) [27]	Population-based	Germany	Community dwellers 70+ ^B^	Suicide thoughts/attempts		Marital status: NS
	Wiktorsson *et al.* (2010) [39]	Clinical	Sweden	Population 70+ ^B^	Suicide attempt		Married/Cohabiting *vs.* not: OR = 0.51 (95% CI = 0.31–0.84)
	Takahashi *et al.* (1995) [37]	Clinical	Japan	Psychiatric inpatients ^B^	Suicide attempt		Marital status: NS
	Tsoh *et al.* (2005) [38]	Nested-Clinical	Hong Kong	Community dwellers ^B^	Suicide attempt/suicide		Marital status: NS
	Conaghan *et al.* (2002) [35]	Clinical	UK	Psychiatric patients and community dwellers ^A^	Parasuicide/depression		Married *vs.* unmarried/widowed: Fisher’s exact = 10.47, *p* < 0.005
	Turvey *et al.* (2002) [41]	Population-based	USA	Community dwellers ^B^	Suicide		Marital status: NS
Living arrangement	Bartels *et al.* (2002) [28]	Clinical	USA	Primary care patients with depression ^C^	Death ideation/Suicidal ideation		Living alone *vs.* living with someone: NS
	Raue *et al.* (2007) [31]	Clinical	USA	Homecare patients ^B^	Suicidal ideation		Living arrangement: NS
	Yen *et al.* (2005) [34]	Population-based	Taiwan	Community dwellers ^B^	Suicidal ideation		Not living alone *vs.* living alone: NS ^iii^
	Dennis *et al.* (2005) [36]	Clinical	UK	Participants with depression ^A^	Self harm behavior		Living alone: NS
	Takahashi *et al.* (1995) [37]	Clinical	Japan	Psychiatric inpatients ^B^	Suicide attempt		Living alone: *p* < 0.01
	Wiktorsson *et al.* (2010) [39]	Clinical	Sweden	Population 70+ ^B^	Suicide attempt		Living alone *vs.* not: OR = 1.90 (95% CI = 1.16–3.11); Living in institution *vs.* not: NS
	Rubenowitz *et al.* (2001) [40]	Psychological autopsy	Sweden	Community dwellers B	Suicide		Living alone *vs.* not—by sex: NS ^ii^
	Waern *et al.* (2003) [42]			Community dwellers 65–75 years. B	Suicide		Living alone *vs.* not: NS
							Residence change: NS ^iv^
				Community dwellers 75+ ^B^	Suicide		Living alone *vs.* not: NS
							Residence change: NS ^iv^
	Tan *et al.* (2008) [33]	Clinical	Singapore	Patients with major depression ^A^	Suicidal thinking		Living arrangement: NS
	Tsoh *et al.* (2005) [38]	Nested-Clinical	Hong Kong	Community dwellers ^B^	Suicide attempt		Living with children: OR = 0.2 (95% CI = 0.03–0.9) ^ii^
							In residential facility: OR = 20.0 (95% CI = 2.5–158.1) ^iv^
				Community dwellers ^B^	Suicide		Living with children: OR = 0.2 (95% CI = 0.04–0.6) ^ii^
	Barnow *et al.* (2004) [27]	Population-based	Germany	Community dwellers 70+ ^B^	Suicide thoughts/attempts		Living in private home/apartment: 1.2%, senior citizens’ home: 0.2%, nursing home: 0.2%. X2 = 4.3, *p* < 0.05
	Fortin *et al.* (2001) [29]	Clinical	Canada	Nursing home residents 69+ ^A^	Suicidal ideation		Months spent in institution: NS
Religion	Bartels *et al.* (2002) [28]	Clinical	USA	Primary care patients with depression ^C^	Death ideation/Suicidal ideation		Frequency of religious activity: NS
	Yen *et al.* (2005) [34]	Population-based	Taiwan	Community dwellers ^B^	Suicidal ideation		Religious affiliation *vs.* not: X2 = 4.03, *p* < 0.05 ^iv^
	Tsoh *et al.* (2005) [38]	Nested-Clinical	Hong Kong	Community dwellers ^B^	Suicide attempt		Ancestor-worshipper: OR = 0.3 (95% CI = 0.1‑0.6) ^iv^
							Religion considered salient: OR = 0.2 (95% CI = 0.1–0.5) ^iv^
				Community dwellers ^B^	Suicide		Religion considered salient: OR = 0.2 (95% CI = 0.1–0.5) ^iv^
	Turvey *et al.* (2002) [41]	Population-based	USA	Community dwellers ^B^	Suicide		Attending religious service at least monthly: OR = 0.29 (95% CI = 0.09–0.83)
Frequency of social contact	Bartels *et al.* (2002) [28]	Clinical	USA	Primary care patients with depression ^C^	Death ideation/Suicidal ideation	Social Network Questionnaire [43]	If any social contacts: Suicide ideation (*vs.* none) OR = 0.86 (95% CI = 0.74–0.99)
	Rowe *et al.* (2006) [32]	Clinical	USA	Homecare patients ^B^	Suicidal ideation	Duke Social Support Index [44]	Social interaction: Suicidal ideation: mean = 5.18, no suicidal ideation: mean = 5.89, *p* < 0.05
	Turvey *et al.* (2002) [41]	Population-based	USA	Community dwellers ^B^	Suicide		At least monthly contact with a child: OR = 1.10 (95% CI 0.38–3.58)
							Presence of a relative they see monthly: OR = 0.65 (95% CI 0.33–1.21)
Low social integration	Dennis *et al.* (2005) [36]	Clincal	UK	Participants with depression ^A^	Self harm behavior (wish to die was reported by most, though not all)	Social Contact Scale [36]	Poorly integrated social network: Self-harm group: 76%, comparisons: 52%, χ^2^ = 5.6, *p* < 0.05
	Tsoh *et al.* (2005) [38]	Nested-Clinical	Hong Kong	Community dwellers ^B^	Suicide attempt	Lubben Social Network Scale [45]	Intensity of low social integration OR = 1.1, *p* < 0.05
				Community dwellers ^B^	Suicide	Lubben Social Network Scale	Intensity of low social integration OR = 1.1, *p* < 0.05
	Rowe *et al.* (2006) [32]	Clinical	USA	Homecare patients ^B^	Suicidal ideation	Duke Social Support Index	Social network size: B = −0.056, SE: 0.042, Wald = 1.790, df = 1, *p* = 0.18
	Yen *et al.* (2005) [34]	Population-based	Taiwan	Community dwellers ^B^	Suicidal ideation	Neighborhood Quality Index [46]	Community participation: with SI = 14.9%, w/out SI = 34.2% (χ2 = 21.2, *p* < 0.001) ^v^
	Rubenowitz *et al.* (2001) [40]	Psychological autopsy	Sweden	Community dwellers ^B^	Suicide		Club/organization member *vs.* not—by sex: NS ^ii^
	Waern *et al.* (2003) [42]						
				Community dwellers 65–75 ^B^	Suicide		Club/organization member *vs.* not: OR = 0.2 (95% CI = 0.1–0.7) ^iv^
				Community dwellers 75+ ^B^	Suicide		Club/organization member *vs.* not: OR = 0.3 (95% CI = 0.1–0.9) ^iv^
Social support	Raue *et al.* (2007) [31]	Clinical	USA	Homecare patients ^B^	Suicidal ideation	Duke Social Support Index	Perceived social support: Any DI/SI in past month: OR = 0.87 (95% CI 0.77–0.98).
	Rowe *et al.* (2006) [32]						Incident DI/SI over one year: OR = 0.80 (95% CI 0.66–0.98)
						Duke Social Support Index	Perceived social support: Suicidal ideation: mean 17.57, no suicidal ideation: mean = 19.27, *p* < 0.01
							Instrumental social support: B = –0.027, SE: 0.065, Wald = 0.169, df = 1, *p* = 0.68
	Turvey *et al.* (2002) [41]	Population-based	USA	Community dwellers ^B^	Suicide		Relative confidant: OR = 0.54 (95% CI 0.29–0.97)
							Friend confidant: OR = 0.41 (95% CI 0.22–0.74)
Loneliness	Wiktorsson *et al.* (2010) [39]	Clinical	Sweden	Population 70+ ^B^	Suicide attempt	Single question: Do you feel lonely?	Feelings of loneliness *vs.* not: OR = 2.8 (95% CI = 1.3–6.1) ^ii^
	Rubenowitz *et al.* (2001) [40]	Psychological autopsy	Sweden	Community dwellers ^B^	Suicide	Recent Life Change Questionnaire [47]	Feelings of loneliness *vs.* not: men: OR = 6.8 (95% CI = 2.6–18.0), women: OR = 8.4 (95% CI = 3.2‑22.3) ^iii^
	Waern *et al.* (2003) [42]						
				Community dwellers 65–75 ^B^	Suicide	Recent Life Change Questionnaire	Feelings of loneliness *vs.* not: OR = 7.6 (95% CI = 2.6–22.3) ^iv^
				Community dwellers 75+ ^B^	Suicide	Recent Life Change Questionnaire	Feelings of loneliness *vs.* not: OR = 5.6 (95% CI = 2.2–14.5) ^iv^
Relationship discord	Rubenowitz *et al.* (2001) [40]	Psychological autopsy	Sweden	Community dwellers ^B^	Suicide	Recent Life Change Questionnaire	Interpersonal conflict within past 24 months: men: OR = 10.0 (95% CI = 1.7–59.8), women: OR = 9.2 (95% CI = 1.97–44.8) ^ii^
	Waern *et al.* (2003) [42]						
				Community dwellers 65–75 ^B^	Suicide	Recent Life Change Questionnaire	Interpersonal conflict: OR = 13.5 (95% CI = 2.7–60.6) ^ii^
				Community dwellers 75+ ^B^	Suicide	Recent Life Change Questionnaire	Interpersonal conflict: OR = 33.7 (95% CI = 3.1–368.5) ^ii^
	Tsoh *et al.* (2005) [38]	Nested–Clinical	Hong Kong	Community dwellers ^B^	Suicide attempt	Life Event Scale [48]	Family discord: OR = 18.0 (95% CI = 2.3–143.3) ^iv^
				Community dwellers ^B^	Suicide		Family discord: OR = 41.0 (95% CI = 5.4–313.6) ^iv^
	Tan *et al.* (2008) [33]	Clinical	Singapore	Patients with major depression ^A^	Suicidal thinking		No relationship difficulties within past month *vs.* relationship difficulties: OR 2.58 (95% CI = 1.01–6.60) ^iii^

Abbreviations: NS: Not significant. ^A^ Study size < 100, ^B^ study size ≥ 100, <1,000, ^C^ study size ≥ 1,000. ^i^ Study participants are aged 65 and above unless otherwise specified. ^ii^ Multivariate regression adjusted for depression and other factors. ^iii^ Multivariate regression. ^iv^ Only confirmed in univariate analyses. NS in multivariate analyses. ^v^ It was furthermore found that “community participation preceding 6-month period was a protective factor from suicidal ideation for those who were male, with religious belief, unemployed, not living alone, with low family income, had physical diseases, and not depressed.” [34].

*Living with family or relative* did not distinguish persons with and without suicidal thinking in the Singapore study by Tan [33]. Living with children was associated with decreased risk for both suicide attempt and death by suicide in the above-cited study by Tsoh [38], and similar odds ratios were reported for both these outcomes. Barnow reported significant associations for both *living in* *nursing homes and living in senior citizens’ homes* in a German population-based study that identified factors associated death wishes/suicidal thoughts/non-fatal suicidal behavior [27]. Institutionalized persons were interviewed in a French-Canadian study by Fortin [29], and no association between number of months spent in the institution and suicidal ideation was found. Living in institution did not increase risk of suicide attempt in the Swedish study conducted by Wiktorsson [39]. Regarding *change of living arrangement*, Tan could show no association with suicide ideation in the Singapore study, though this question was based on the previous four weeks only [33]. Residential change over the past two years was not associated with death by suicide in the Swedish study by Rubenowitz [40], nor when Waern conducted separate analyses for those aged 65–74 years and those aged above 75 [42].

Four studies (two from the U.S., one from Hong-Kong and one from Taiwan) examined aspects of *religiosity* [28,34,38,41]. Two of these addressed suicidal ideation. No association could be shown between frequency of religious activity and either death ideation (*i.e.*, wishes for death) or suicide ideation in primary care patients in the U.S.-based study by Bartels [28]. In a population-based study set in Taiwan, Yen found that having no religious affiliation was more common among those with suicidal ideation than in those without [34]. The acknowledgement of the importance of religious beliefs was associated with reduced risk of both non-fatal and fatal suicidal behavior in the Hong Kong study by Tsoh [38]. Finally, Turvey [41] reported that persons in the U.S. who died by suicide were less likely to attend religious services at least monthly compared to a community-based comparison group. 

Several U.S. studies (n = 3) examined *frequency of social contact*, with studies by Bartels and Rowe documenting associations between limited social contact and suicidal ideation [28,32] and a study by Turvey showing an association with suicide death [41]. However, when specifically considering contacts with children and relatives, null results were found for death ideation (*i.e.*, passive wishes for death) in the Bartels study [28] and for suicide in the Turvey study [41]. 

Indices of *low social integration* were examined in five studies. The studies varied in the type of social integration measure that was used. Two studies used composite measures of social integration that include information about quality and quantity of people in subjects’ social networks. For these two studies, significant effects on non-fatal suicidal behavior were observed in the Dennis study from Great Britain [36] and on both attempted suicide and suicide in Tsoh’s Hong Kong-based study [38]. Rowe examined a single aspect of social integration—the size of the social network in homecare patients in the U.S.—and did not find an effect on suicidal ideation [32]. Finally, two studies examined another aspect of social integration—*community participation*. Yen noted that older adults in Taiwan who reported suicide ideation were statistically significantly less likely to report that their neighborhood was characterized by community participation [34]. Decreased suicide risk was observed for Swedish men and women who participated in organizations in the study by Rubenowitz [40]. When stratified analyses were carried out, Waern noted that both younger (65–74) and older (75+) suicide decedents were significantly less involved in such activities than sex- and age-matched comparisons from the general population [42].

Two studies (across three publications) explored aspects of *social support*. Raue and Rowe examined both instrumental and perceived social support in a prospective study of older adults receiving home nursing services [31,32]. Instrumental support did not differentiate older adults with and without death/suicide ideation [32]. However, greater perceived social support was associated with lower risk for both concurrent and one-year incident death/suicide ideation [31]. Turvey reported that the presence of a relative or friend who was thought of as a confidante was associated with decreased likelihood of suicide [41]. In that study a confidante was defined as someone the older adult felt close to and could talk to about private matters (*i.e.*, a provider of social support). 

*Loneliness*—the inner, subjective experience of social disconnectedness—was examined in two Swedish studies (across three publications), documenting increased risk for both non-fatal and fatal suicidal behavior [39,40,42]. The presence of loneliness was associated with nearly three times greater risk of a suicide attempt in the study by Wiktorsson [39]. Rubenowitz reported that loneliness was associated with increased risk of suicide in both men and women [40]. Further, when Waern [42] analyzed data separately for those aged 65–74 years and those aged above 75, loneliness was found to be a stronger predictor among the latter age group.

An index of discordant connectedness—*relationship discord*—was examined in three publications, documenting associations with non-fatal [38] and fatal suicidal behavior [38,40,42]. Analysis of data from the Swedish psychological autopsy study revealed that interpersonal conflict over the prior 24 months was a significant predictor of suicide [40,42]. The Tsoh study [38]—described above in the section on social integration—did not find that family discord distinguished between suicide attempts and deaths, but it did distinguish between community controls and both attempts and deaths. In the Singapore-based study by Tan an *absence* of relationship difficulties was associated with greater likelihood of reporting suicidal ideation, even after adjusting for whether the older adults lived alone [33].

## 4. Discussion

This systematic review yielded 16 publications on social factors and suicidal behaviors in persons aged 65 and above. These studies were based on data from 14 studies set in three continents (U.S. and Canada n = 6, Europe (Germany, Sweden, and United Kingdom) n = 4, and Asia (Hong Kong, Japan, Singapore, Taiwan) n = 4). Before discussing the results, some issues related to methods need to be noted. Inclusion age was 65 years and older and only studies that employed similar age comparison groups were included. The rationale for this was that rates of suicidal behavior and social factors associated with such behavior vary widely by geographic region. We wanted to identify studies that provided odds ratios for this specific age group. We included marital status and living arrangements in our review because these factors can help to define the target population. Participation in religious activities was also included, as religious centers may in some settings provide important occasions for social interaction. 

Our review has a number of limitations. We included a broadly defined range of suicidal behaviors (death wishes, suicide ideation, non-fatal and fatal suicidal behavior). It cannot be assumed that these phenomena share the same risk factors. Further, a meta-analysis was not feasible given the limited number of publications, and the broad spectrum of recruitment procedures, variables of interest, and instruments employed. A final caveat is that, with the exception of the study by Rubenowitz and colleagues [40], sex-specific data for older adults were lacking. This is particularly problematic considering the vast sex differential in suicide rates observed among older adults in most industrialized countries for which suicide data are available [20]. Although there is evidence of gendered norms of suicidal behavior in countries with large sex differentials (for example, in the United States, non-fatal suicidal behavior is considered feminine while suicide is more acceptable in men than in women [49]), more research is needed to elucidate the details of cultural scripts of gender and suicidal behavior within and between countries. It is probable that gendered beliefs and experiences play a role in the association between social factors and suicide [20,50], just as there are gender-related differences in the link between physical illness and suicide [51].

The majority of the social factors examined in this review can be conceptualized as indices of social connectedness—the degree of positive connectedness to family, friends, and social groups. However, the most often-studied factors—marital status, living arrangements, and frequency of social contact—yielded the most inconsistent results across studies (see Table 2). Heterogeneity across studies may be attributed to the fact that such factors are poor indicators of the subjectively experienced degree of social connectedness. This apparent heterogeneity is systematically moderated by macro- or national-level influences, for example, cultural scripts of gender and suicidal behavior [49] that we did not examine. 

**Table 2:** Box score summary of review findings.

Variable	Number of studies	Some evidence of association with outcome		
		Yes	Mixed	No
Marital status	11	4		7
Living arrangement	11	3	1	7
Religion	4	3		1
Frequency of social contact	3	1	2	
Low social integration	5	4		1
Social support	2	1	1	
Loneliness	2	2		
Relationship discord	3	3 ^A^		

^A^ One study found that *absence* of relationship discord was associated with suicidal ideation [33].

Most of the studies that examined marital status did not show an association with suicidal behavior. While being married is often associated with better health outcomes, especially for men, marital status *per se* tells us nothing about the quality of the relationship. Even marriages that have lasted for decades can come under strain when age-related changes affect one or both of the partners. Loss of mental and physical health and functional ability are examples of factors that can vastly change circumstances for a married couple. Bereavement is common in later life, and there is evidence that risk of suicide is particularly heightened in older men who have lost a spouse. In a population-based register study of the entire Danish population aged fifty year and older, Erlangsen and colleagues showed that men aged 80 years and above who had lost their partner during the past year had a 15-fold increase in suicide risk compared to married middle-aged men [52]. 

Only two of the eleven studies that examined living arrangement reported an association between living alone and the outcomes of interest, one from Japan [37] and another from Sweden [39]. The variable living alone simply designates that the person is the sole resident in the dwelling; it does not capture the surrounding social circumstances. The living alone variable could be confounded by other factors. For example, those who live with their children would belong to the group who do not live alone, and living with children was associated with a decreased risk for both non-fatal and fatal suicidal behavior in the Hong Kong study [38]. Further, a person with a limited social network might be more likely to require institutional care, whereas one with a large social network might receive support that enables continued living at home. 

Rather consistent results were found in this review for religious participation, with four analyses across three studies from Taiwan [34], Hong Kong [38], and the U.S. [41] documenting inverse associations with ideation, non-fatal and fatal suicidal behavior. Two U.S.-based studies focusing on persons aged 50 and above found that those who died by suicide less frequently took part in religious activities, compared to those who died of natural causes [53,54]. One of these [54] demonstrated that those who did participate in religious activities had a less active role in the congregation compared to their counterparts. Another study that focused on medically ill older persons found that church attendance and private religious activities did not predict shorter time to remission of depression, but religious beliefs (intrinsic religiosity) did [55]. Religious activities often provide an important channel for social interaction, even for those who cannot physically attend services. A functionally impaired older adult may find a sense of connectedness to the religious community, for example through radio or other media, or through home visits by clergy or congregation members. This may be particular important for older men, given men’s less developed connectedness with family, relative to older women [20].

Three of the five analyses of frequency of social contact (across 3 studies, all from the U.S. [28,32,41]) yielded significant associations between low contact and both suicide ideation and suicide. An additional case-control New Zealand study with a lower inclusion age (55+) [56] also documented an association between low social contact and suicide, but a Canadian study (60+) that examined contacts during the last six months showed no difference between suicide decedents and control subjects [57]. No associations were observed in the studies reviewed when social contacts included only family, tentatively suggesting that contacts with friends may be particularly protective. Most studies documenting significant effects used “less than monthly contact” as the indicator of limited social contact. A more “severe” indicator of infrequent social contact (*i.e.*, no contact in the previous year) was used in a British study [58] that compared older adults (60+) who died by suicide to those who died in hospital by natural causes. While the proportion with less than daily contact with friends/relatives in the year before death was numerically smaller in the suicide group, no difference could be shown. Of course the risk for premature mortality conferred by infrequent social contact is not specific to suicide [59], though the strength of the association, as well as underlying mechanisms, may differ as a function of type of health outcomes (e.g., cancer mortality, as compared to suicide mortality). 

Three analyses across two independent studies, one from Great Britain [36] and another from Hong Kong [38], established a consistent association between low social integration and fatal and non-fatal suicidal behavior. Further, the results of these studies suggest that the effect is not due solely to depression. Though investigated in fewer studies, consistent results were also found for subjective measures of social connectedness—perceived social support (two U.S. studies [31,41]) and loneliness (two Swedish studies [39,40,42]). Low instrumental support, in a single U.S. study [31], was not associated with suicide. Reasons for this were not examined. Other research indicates stronger benefits for giving versus receiving support [60]. Further, there are findings indicating associations with perceiving oneself as a burden and suicide risk in later life [16]. Taken together, these findings suggest that instrumental support should be measured separately from other types of social support, such as emotional support and companionship, because some specific aspects of social support may mitigate risk of suicidal behavior. 

Qualitative studies can provide insights on individuals’ perceived social connectedness- or lack thereof. A recent Norwegian psychological autopsy study used a qualitative approach to explore relationships between the informants and the older adult (65+) who died by suicide. Findings showed that the relationships were often difficult and distant [61]. In a qualitative study set in Britain, participants (aged 65 and above) described feeling disconnected to their surroundings prior to their suicidal act. For some, successful coping after the act meant becoming more connected and more visible to others [62]. 

## 5. Future Research

The need for future research is clearly identified by the fact that our broad inclusion criteria yielded a limited number of studies to be reviewed. As populations age, it will be increasingly important to elucidate risk estimates for social factors associated with suicidal behaviors also in oldest old populations. Further, studies need to be carried out in different settings throughout the world, in order increase understanding of how societal and cultural conditions may shape the influence of social factors on suicide. The relevance of Durkheim’s theories remains to be elucidated with regard to suicidal behavior in older persons. 

Future research may include assessment of subjective accounts of social connectedness (*i.e.*, perceived social support, loneliness), as well as social integration and participation (*i.e.*, complex social integration measures and participation in clubs). Qualitative studies with a focus on experiences of social connectedness/thwarted belongingness in older persons with non-fatal suicidal behavior could provide important knowledge that quantitative studies cannot provide. In the studies using the psychological autopsy approach, this will require asking close informants to provide data pertaining to the decedent’s subjective experience of loneliness. Much has been learned over the past decade about the capacity of informants to provide “accurate” data about the internal state of another person [63,64], including in older adults [65,66]. For example, it has been shown that informants are likely to over-estimate self-consciousness and to underestimate the extent to which someone experiences positive emotions. Some of these discrepancies can be ascribed in part to the person’s level of cognitive function or medical burden [66]. These and similar findings could inform the next generation of psychological autopsy research. 

To date, relatively few studies of elderly and suicidal behavior have included questions regarding religiosity, as shown in this review. The multidimensionality of religion makes the phenomenon difficult to study. However, assessment of the individual’s religiosity should be considered in future studies. The impact that culture and gender has on religion and its impact on suicidal behavior needs to be taken into consideration in future research [67]. 

A life course perspective, one that considers longstanding personality traits known to influence suicide [68,69] as well as age related changes in social roles and networks, can provide further understanding. Some suicidal older persons may have had a rich social network that became restricted in the context of aging. For others, lack of social connectedness may have been a more enduring experience, perhaps related to personality [70]. Results from a qualitative study focusing on U.S. older women showed that those who were suicidal experienced loneliness in childhood, and no confidants during adolescence and adulthood [71]. 

A neuropsychological perspective may also provide further insight into suicidal behavior among older adults. A recent U.S. study that focused on depressed **s**uicide attempters aged 60 and above found that social cognition deficits differentiated individuals who had engaged in suicidal behavior from mentally healthy controls [72]. Specifically, individuals in the former group demonstrated significantly more errors on the Reading the Mind in the Eyes test, which measures emotion recognition capabilities by having participants make subtle discriminations between photographs of facial expressions. As indicated by the authors, difficulties with emotion recognition could impair social problem solving, lead to conflict in social interactions, and result in lower feelings of social connectedness and belonging.

Theoretical perspectives are also relevant and may generate directions for future research. The Interpersonal Theory of Suicide [16] proposes that individuals have intrinsic needs to belong to meaningful relationships and groups. If this need is thwarted, and the individual has a perception of being a burden on others, this will result in a desire for suicide. Recent U.S. research examining the theory with older adults indicates that perceived burdensomeness is associated with more severe suicidal ideation above and beyond the contribution of belongingness [73,74]. Thus, interventions that alleviate feelings of thwarted belongingness and burdensomeness are relevant to late-life suicide prevention in the U.S. Finally, progress in understanding late life suicidal behavior requires attention to its cultural meanings [75]. The cultural script theory of suicidal behavior [75] points to ways to understand the gender gap in older adult suicidal behavior found in many industrialized countries via a focus on gendered meanings of suicidality as well as on the gendered experiences of social connectedness in these countries [20], with implications for prevention. 

A number of carefully designed, multidisciplinary, prospective studies of older adult cohorts implemented in the 1970’s continue to yield insights into suicide risk [76,77,78]. A new generation of large-scale prospective studies can broaden our knowledge of the determinants and course of social connectedness and the development of suicidal behavior in older individuals over time, which can help to inform appropriate interventions.

## 6. Implications for Suicide Prevention

Controversy abounds concerning the labels used to designate suicide prevention research. Several typologies have been proposed [79,80,81,82]. Distinctions have been drawn between high-risk approaches and population-based approaches [79,80] and between universal, targeted, selective, and indicated approaches [81,82]. For our purposes, a key issue is whether or not programs target populations on the basis of a clinical risk factor (depression, suicide ideation, non-fatal suicidal behavior). Following Kaplan [79], these two approaches are labeled secondary prevention and primary prevention. 

Guided by a medical model of health, secondary prevention targets the clinical conditions and diseases known to confer suicide risk (e.g., major depression). Ideally, this would involve determining whether an evidence-based treatment offered to patients with a clinical condition (such as major depression with suicide ideation and feelings of loneliness) leads to fewer suicides over a follow-up period than a control treatment. Research indicates that indices of social connectedness are modifiable among older adults using social interventions, including group psychotherapy [83], art/recreational therapy [84,85,86,87], and peer support [88]. Among middle-aged adults, cognitive behavior therapy has also been shown to be effective [89]. Interpersonal psychotherapy has been shown to reduce suicide ideation among older adults [90]. Further, primary care providers may wish to consider the implications of their practice patterns for their patients’ experiences of care and connectedness. Office space, the behavior of office staff, and provider interviewing techniques may all affect patients’ experiences of care and being cared for.

Major problems in suicide prevention are the paucity of specialized mental health services and the reluctance of many at-risk people, especially men, to avail themselves of treatment due to stigma [91] and social norms [92]. Primary prevention may be particularly useful in mitigating risk among individuals, who, for whatever reason, do not access mental health services. Guided by a behavioral model of health, the main targets of primary prevention programs are typically health behaviors and health decision-making [79]: alcohol, tobacco, drug use, maintenance of a healthful diet, exercise, and safety (with regard to sexual behavior, food, medication, transportation, firearms). Primary prevention programs of greatest demonstrable relevance to suicide in communities where firearms and medications are dominant suicide methods include legislation restricting access to unsafe firearms as well as mandates concerning the manufacturing, packaging, and distribution of medications [93,94]. Public health messaging interventions designed to modify attitudes about aging, the receipt of mental health services, and the acceptability of suicide might also influence suicide rates. Primary prevention programs designed to enhance positive social connections between neighbors as well as a sense of community might also decrease suicide risk. These programs may prove to be particularly effective because they do not require people to make an active choice (to see a doctor, take a medication), and typically do not threaten to undermine individual autonomy [95]. Further, these programs can reduce a host of negative outcomes beyond suicide. Such programs would involve collaborations among social scientists, physicians, community leaders, including religious leaders, and experts in environmental planning and design. 

## 7. Conclusions

Findings of this review of the literature on social factors in older adult suicidal behavior suggest that limited social connectedness is associated with suicidal ideation, non-fatal suicidal behavior, and suicide in later life, at least in industrialized countries. Primary prevention programs designed to enhance social connections as well as a sense of community could potentially decrease suicide risk. Such programs would involve collaborations among social scientists, physicians, community leaders including religious leaders, and experts in environmental planning and design.
